# Inversion of high-amplitude magnetic total field anomaly: an application to the Mengku iron-ore deposit, northwest China

**DOI:** 10.1038/s41598-020-68494-1

**Published:** 2020-07-20

**Authors:** Jianhua Yang, Shuang Liu, Xiangyun Hu

**Affiliations:** Hubei Subsurface Multi-scale Imaging Key Laboratory, Institute of Geophysics and Geomatics, China University of Geosciences, Wuhan, China

**Keywords:** Geophysics, Solid Earth sciences

## Abstract

In magnetic prospecting, the total field anomaly formula that represents the projection of the magnetic anomaly vector on the geomagnetic field is widely used because it simplifies the calculation of forward modelling and inversion of magnetic data. However, the projection anomaly yields errors relative to the true observed magnetic anomaly, especially for high-amplitude magnetic anomalies such as in iron orebody and unexploded ordnance prospecting. In this study, we analyse the difference between the projection anomaly and observed modulus difference anomaly with physical parameters, and propose to directly invert for the modulus difference anomaly by constructing a nonlinear matrix equation between the model corrections and data corrections. The inversion is then implemented using a preconditioned conjugate gradient algorithm. Synthetic and field magnetic data were used to test the inversion method. Comparison of the two types of total field anomalies shows that the error of the projection anomaly increased with increasing total-field magnetic anomaly. When the total-field magnetic anomaly was < 5,000 nT, the difference between the projection anomaly and modulus difference anomaly results can be ignored. For high-amplitude magnetic anomalies, the modulus difference anomaly inversion produced more accurate representations of both the shape and location of the magnetic sources.

## Introduction

Magnetic anomalies are caused by diversities between the magnetic properties of the target body and the surrounding lithosphere. This difference in magnetic properties can be applied in geophysical exploration such as edge detection^1,2^, mineral^3–5^, unexploded ordnance (UXO)^6–8^ exploration, and geological investigation^9–15^. Magnetic anomaly surveying is a most effective tool for mineral exploration. One of the main issues in mineral exploration is the precision of the data processing and interpretation. In recent years, many strategies have been proposed for the stage of high-precision and quantitative data-processing, inversion and interpretation in mineral exploration data^16–22^. However, the anomaly in traditional methods is the projection of the magnetic anomaly vector on the normal geomagnetic field. It is seen as the approximation of observed modulus difference anomaly. Generally, the traditional method uses the projection anomaly to replace the modulus difference anomaly for data-processing and interpretation when the total-field magnetic anomaly is less than 5,000 nT, and the difference between projection anomaly and modulus difference anomaly can be neglectable^23–26^. The projection anomaly can be seen as a component of the magnetic anomaly vector. Then, this assumption makes projection anomaly has a physical meaning in a specific direction in contrast to the modulus difference anomaly. It provides a theoretical basis for the transformation between the projection anomaly and the other three fixed direction components of the magnetic anomaly vector. The relation between the projection anomaly and the physical properties of magnetic bodies can be transformed into a linear matrix-equation, which simplifies the forward and inversion process and improves efficiency.

The projection anomaly and the modulus difference anomaly are two factors that have similarities and differences. One of the main differences between them can be seen in high-amplitude magnetic anomalies. Yuan et al.^27^ found that the higher the magnetic anomaly, the larger the errors through 2-D numerical experiments with high-amplitude anomalies. It demonstrates that projection approximation yielded errors in strong magnetic environments, which is clearly shown and cannot be ignored in data analysis. Zhen et al.^28^ analysed the relative-error (the difference/the magnitude magnetic anomaly) reach 10% with spherical model. They suggest projection anomaly is not credible especially as high-precision of quantitative processing and interpretation are required and propose a method using the error yielded by the projection anomaly. Coleman and Li^29^ studied the difference between the errors of the total-field anomaly and magnetic amplitude data and found that the errors in the three orthogonal components converted from the total-field anomaly had similar standard deviations. Based on the total magnetic anomaly is no longer approximately equal to projection anomaly in the highly magnetic environments, Sun et al.^30^ used the information from the borehole data and structural orientation as constraints and get an acceptable result. In our study, we define the error between projection anomaly and observed modulus difference anomaly firstly and carefully discuss the error caused by amplitude, inclination and declination individually and together between the projection anomaly results (data processing, inversion and interpretation) with those of the modulus difference anomaly for strong magnetic anomalies. Second, we propose directly inverting for the modulus difference anomaly in strong magnetic bodies under Cartesian coordinate. Third, the operator, given by modulus difference anomaly, is nonlinear which is different with projection anomaly. Finally, the synthetic and field data are used to test the inversion method with two types of total field data.

## Total field anomalies

### Two types of total field anomalies: projection anomaly and modulus difference anomaly

**Figure 1:**
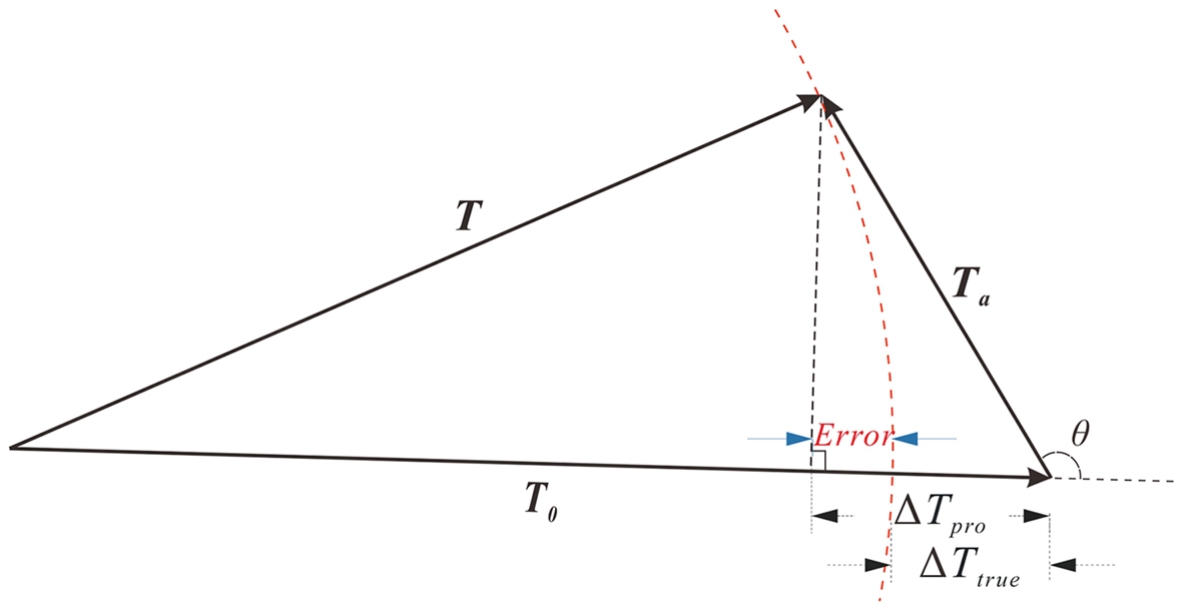
Sketch of the projection anomaly (Δ*T*_pro_) and modulus difference anomaly (Δ*T*_true_) showing the geometry with relation to the observed anomaly **T**, magnetic anomaly vector **Ta** and the normal geomagnetic field **T**_**0**_.

Generally, the obtained anomaly **T** at any point is the vector sum produced by all the magnetizations, comprising two contributions: the normal geomagnetic field **T**_**0**_ and the magnetic anomaly vector **T**_**a**_ (Fig. 1). The modulus difference anomaly (Δ*T*_*true*_) is the modulus difference between **T** and **T**_**0**_:

1 $$\Delta T_{{_{true} }} = \left| {\mathbf{T}} \right| - \left| {{\mathbf{T}}_{{\mathbf{0}}} } \right|$$

and

2 $${\mathbf{T}}_{a} = {\mathbf{T}} - {\mathbf{T}}_{{\mathbf{0}}} .$$

Experimental data^31–33^ confirmed that when the magnetic amplitude data *T*_*a*_ is far less than *T*_*0*_, the magnetic anomaly can be simplified for forward modelling. In this way, the modulus difference anomaly can be regarded as the projection anomaly (Δ*T*_*pro*_). To compare the difference between the modulus difference anomaly and projection anomaly, we define the error as:

3 $$Error = \Delta T_{true} - \Delta T_{pro} { = }\left( {\sqrt {T_{0}^{2} + T_{a}^{2} + 2T_{0} T_{a} \cos \theta } - T_{0} } \right) - T_{a} \cos \theta .$$

where *θ* is the angle between the normal geomagnetic field and the magnetic anomaly vector. The Eq. (3) demonstrates that the error arises mainly from the value of the magnitude magnetic anomaly and the angle *θ*.

## Comparison of the projection and modulus difference anomalies

We first test the method through the cube model, which has a length of 100 m and a central burial depth of 100 m. The horizontal coordinate of the centre of the model is (500, 500). The geomagnetic field *T*_*0*_ is 50,000 nT, the total inclination *I* is 45° and the total declination *D* is 0°. The magnetic anomalies can be changed by changing the magnetization intensity (*m*) of the model.

**Figure 2:**
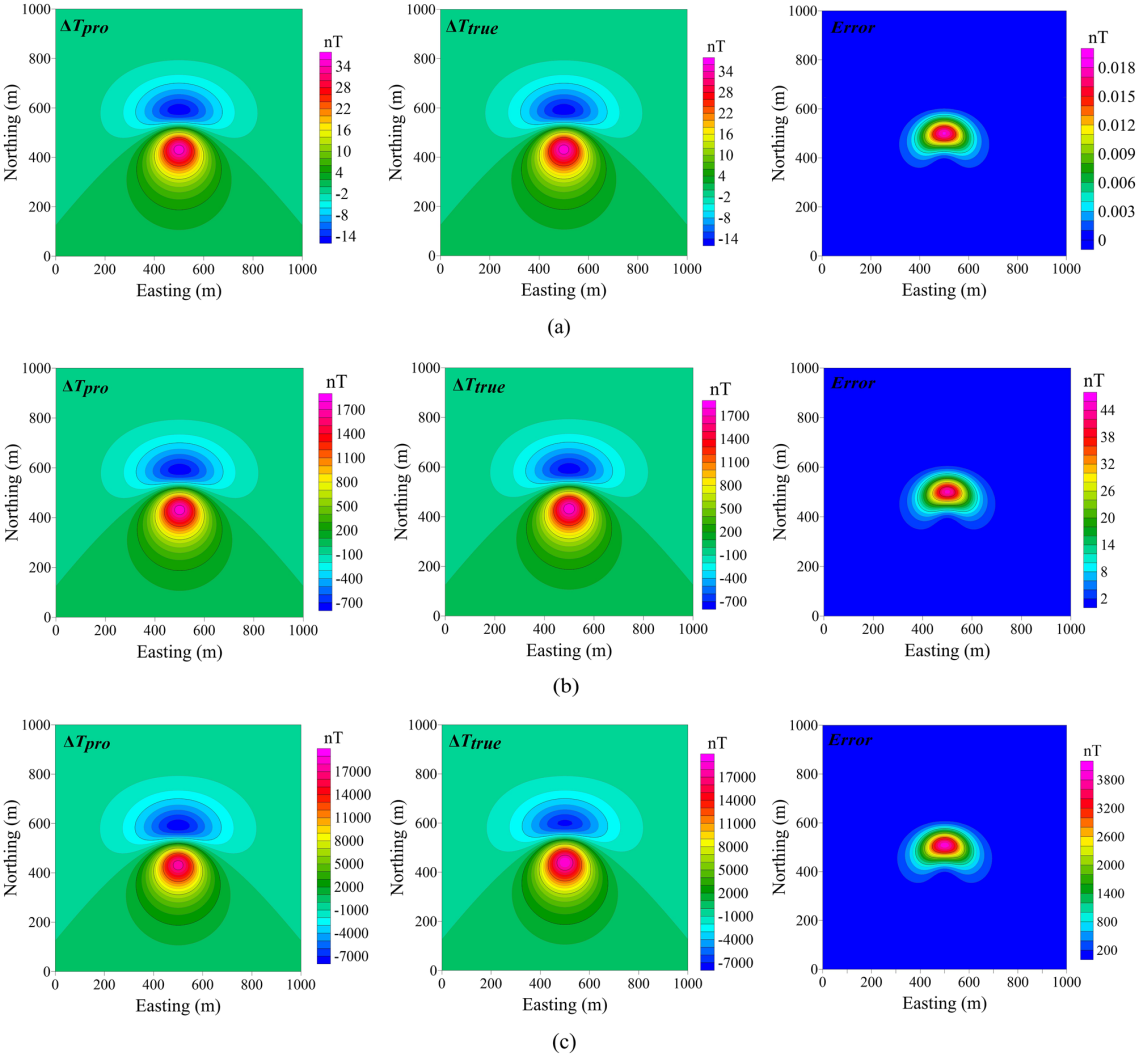
Comparison of two different types of total field anomalies produced by a cube model when the magnetization intensity is (**a**) 1 A/m, (**b**) 50 A/m and (**c**) 500 A/m. The left, middle and right panels show the projection anomaly, modulus difference anomaly, and their difference, respectively.

The projection anomalies, modulus difference anomalies and errors for *m* = 1, 50 and 500 A/m are shown in Fig. 2. When *m* = 1 A/m (Fig. 2a) or *m* = 50 A/m (Fig. 2b), the magnetic anomalies are less than 5,000 nT and the differences between Δ*T*_*pro*_ and Δ*T*_*true*_ are small. Additionally, the magnetic anomalies calculated by these two methods are very close in shape and value. When Δ*T*_*true*_ = 1,794 nT and Δ*T*_*pro*_ = 1,783 nT, the maximum error is 45 nT, which is less than 2% of the total-field magnetic anomaly (Fig. 2b). In this case, the error that arises from using Δ*T*_*pro*_ to replace Δ*T*_*true*_ is small and may be neglected. However, when *m* = 500 A/m (Fig. 2c), Δ*T*_*true*_ = 18,920 nT and Δ*T*_*pro*_ = 17,830 nT, which results in a big difference between them. The maximum error at this time is 3,945 nT, accounting for 21% of the total-field magnetic anomaly’s peak value.

**Figure 3:**
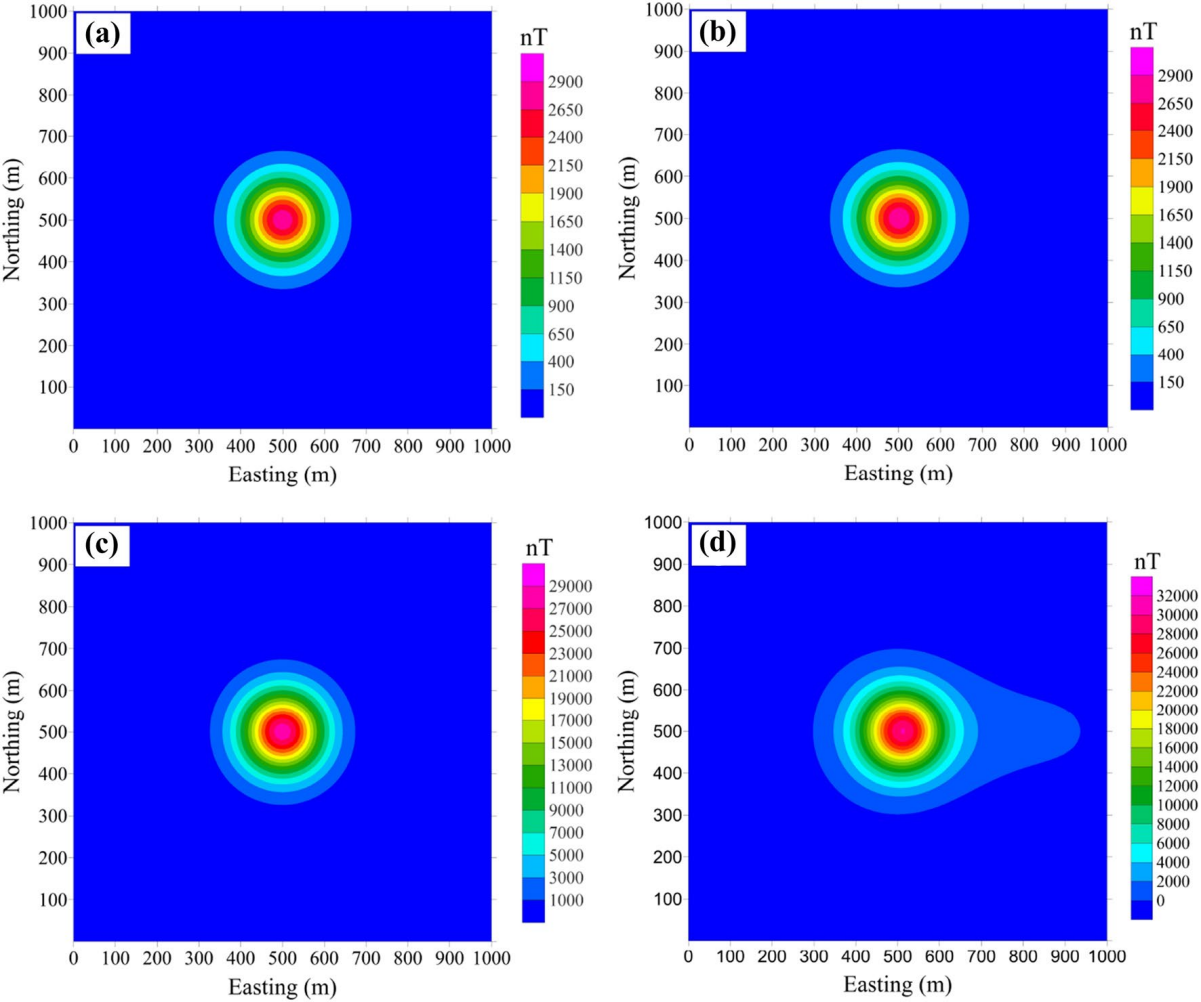
Computed RTP anomaly from the projection anomaly of the cube model for magnetization intensities of (**a**) 50 A/m and (**c**) 500 A/m; the computed RTP anomaly from the modulus difference anomaly of the cube model for magnetization intensities of (**b**) 50 A/m and (**d**) 500 A/m.

In Hinze’s research^24^, the error can reach 1,667 nT as *T*_*0*_ = 30,000 nT and *T*_*a*_ = 10,000 nT, which is close to our numerical simulation result. Such a big error (3,945 nT) has a deep negative impact on the interpretation and even absolutely mistake. The error between Δ*T*_*pro*_ and Δ*T*_*true*_ should be considered carefully when the data indicate high magnetic amplitudes^30^. To understand the difference between Δ*T*_*pro*_ and Δ*T*_*true*_, we converted the magnetic anomalies to reduction to the pole (RTP) values for *m* = 50 A/m (Fig. 3a, b) and *m* = 500 A/m (Fig. 3c, d). The RTP result of real data (Fig. 3d) cannot eliminate the asymmetry of magnetic anomalies caused by magnetization field, which is unacceptable for interpretation. Both the amplitude and shape of the RTP results in the strong magnetic environments were significantly different (Fig. 3c, d). The error is so obvious that projection anomaly is no longer suitable for interpretation of high-precision measurements. This indicates that Δ*T*_*pro*_ cannot be regarded as equal to Δ*T*_*true*_ in strong magnetic bodies because the error between them will be further enlarged during data processing and interpretation.

**Figure 4:**
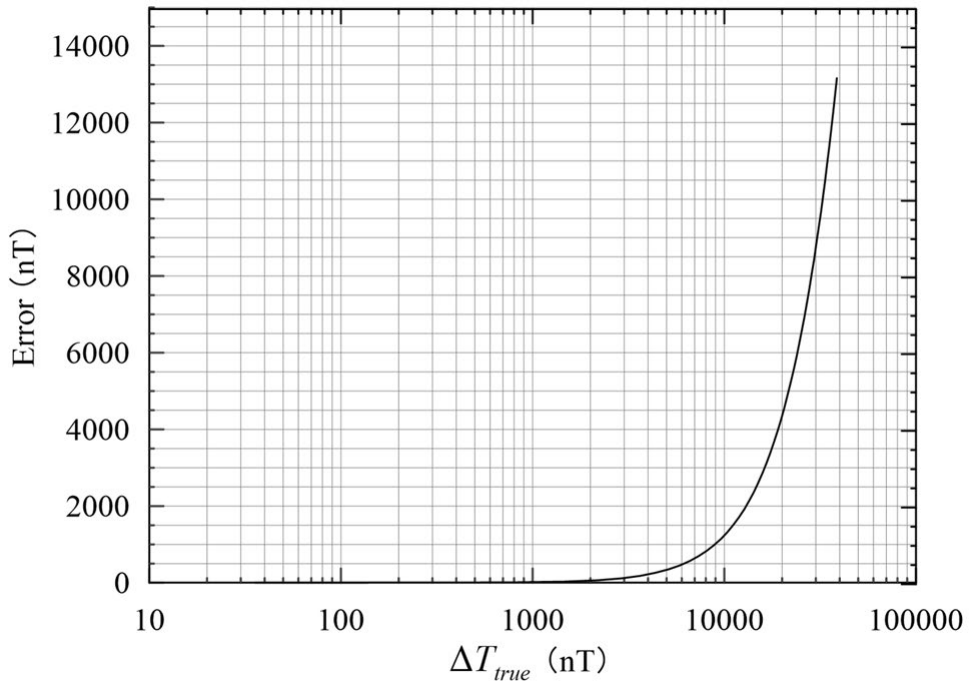
Difference between the projection anomaly and modulus difference anomaly for magnetic anomaly amplitudes of 0–100,000 nT.

Figure 4 shows the error (Δ*T*_*true*_ − Δ*T*_*pro*_) for observed modulus difference anomaly with different amplitudes. When the total-field magnetic anomaly is less than 1,000 nT, the maximum error is less than 10 nT and can be ignored. When the total-field magnetic anomaly reaches 2,000 nT, the maximum error is 54 nT (see *Error* in Table 1). As the magnetic anomaly increases to 5,000 nT and 10,000 nT, the maximum error is close to 335 nT and 1,301 nT, respectively (see *Error* in Table 1). It is far beyond the precise magnetic allowance. What’s more, the error increases exponentially as the total-field magnetic anomaly rises and the order begins to approach the order of the total-field magnetic anomaly. For magnetic bodies of high susceptibility, the real data (observed modulus difference anomaly) have a high amplitude in ground or borehole magnetic surveys^34,35^, especially for shallow ores bodies, which is more than 10,000nT. When we use projection anomaly to fit the real data approximately, it will do harm to exploration resource. For instance, it may lead to spending more time and money on drilling to verification, which causes the waste of resource.

**Table 1:** Maximum error with different magnetic anomaly values.

The amplitude of magnetic anomalies (nT)	*Error* (nT)	*E* (nT)
100	/	0.1
1,000	13	10
2,000	54	40
5,000	335	250
10,000	1,301	1,000

**Figure 5:**
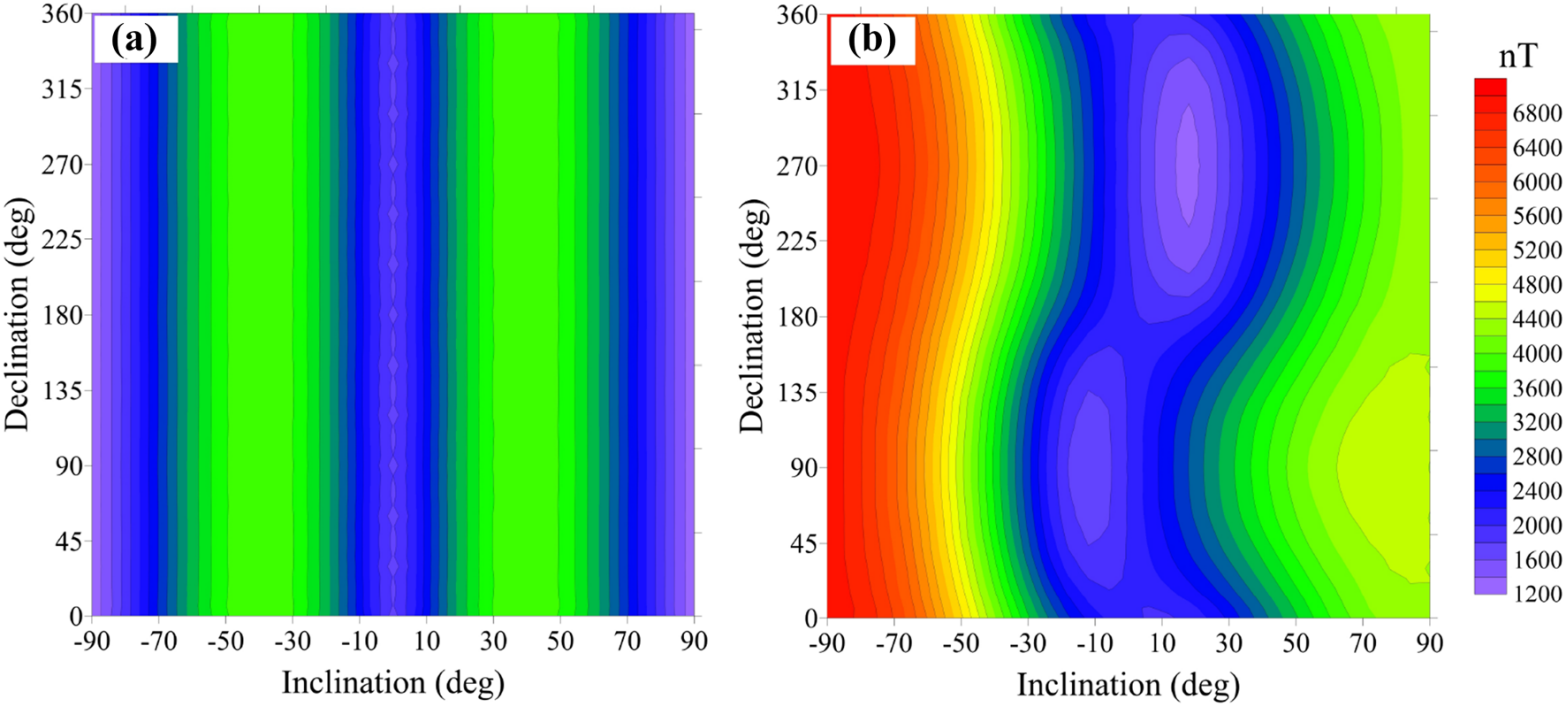
Difference between the projection anomaly and modulus difference anomaly for magnetic inclination values from − 90° to 90° and a magnetic declination values of 0–360°; (**a**) with induced magnetization directions and (**b**) with remanent magnetization directions.

According to Eq. (3), the angle *θ*, between the magnetic anomaly vector **T**_**a**_ and the geomagnetic field **T**_**0**_, is also a factor affecting the error. The angle *θ* depends mainly on the magnetic inclination (*I*) and declination (*D*). Therefore, we looked at the error (Δ*T*_*true*_ − Δ*T*_*pro*_) caused by the magnetic direction for magnetic inclination values from − 90° to 90° and a magnetic declination range of 0°–360°. Figure 5a shows the error distribution calculated for Δ*T*_*true*_ and Δ*T*_*pro*_ without remanence. The amplitude of the total field magnetic anomaly is 28,500 nT and the maximum error is 3,992 nT, which is 14% of the total field magnetic anomaly. The influence of the magnetic declination is negligible and the error distribution has good symmetry. When the error is calculated for data with the magnetic remanence (Fig. 5b; *I*_*0*_ = 45°, *D*_*0*_ = 0°), the amplitude of the magnetic anomaly is 24,721 nT and the maximum error is 6,935 nT, which is 28% of the total field magnetic anomaly. The error under the influence of remanence is almost one time bigger than those without remanence. There is no doubt that the difference between Δ*T*_*true*_ and Δ*T*_*pro*_ is enlarged under the influence of remanence. In practice, most of magnetic ores are affected by remanence. So it is high time that we should take the impact of approximation by projection anomaly in data process into account.

**Figure 6:**
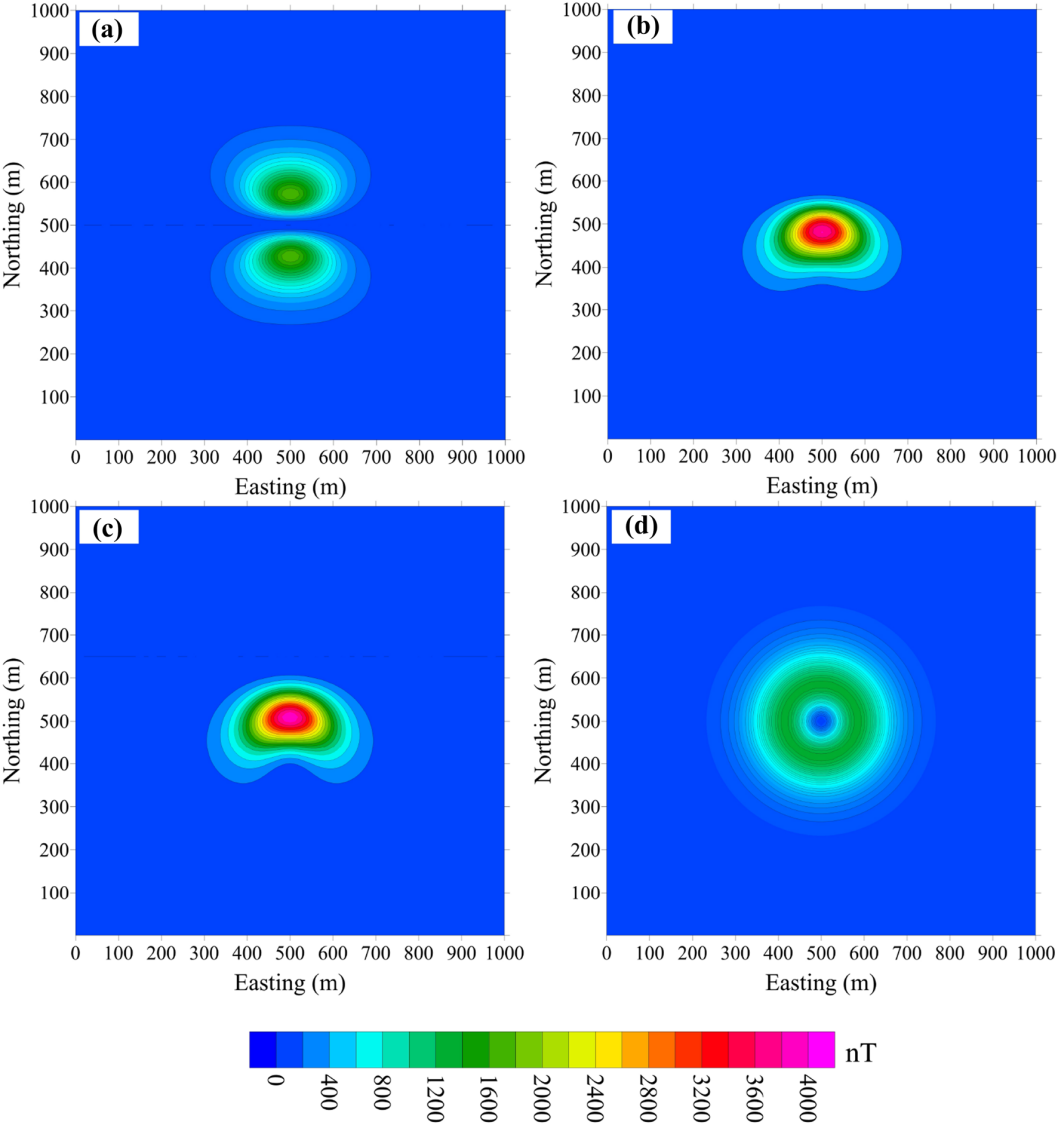
Difference between the projection anomaly and modulus difference anomaly without remanence, where the purely induced magnetization inclination is equal to (**a**) 0°, (**b**) 30°, (**c**) 45° and (**d**) 90°.

**Figure 7:**
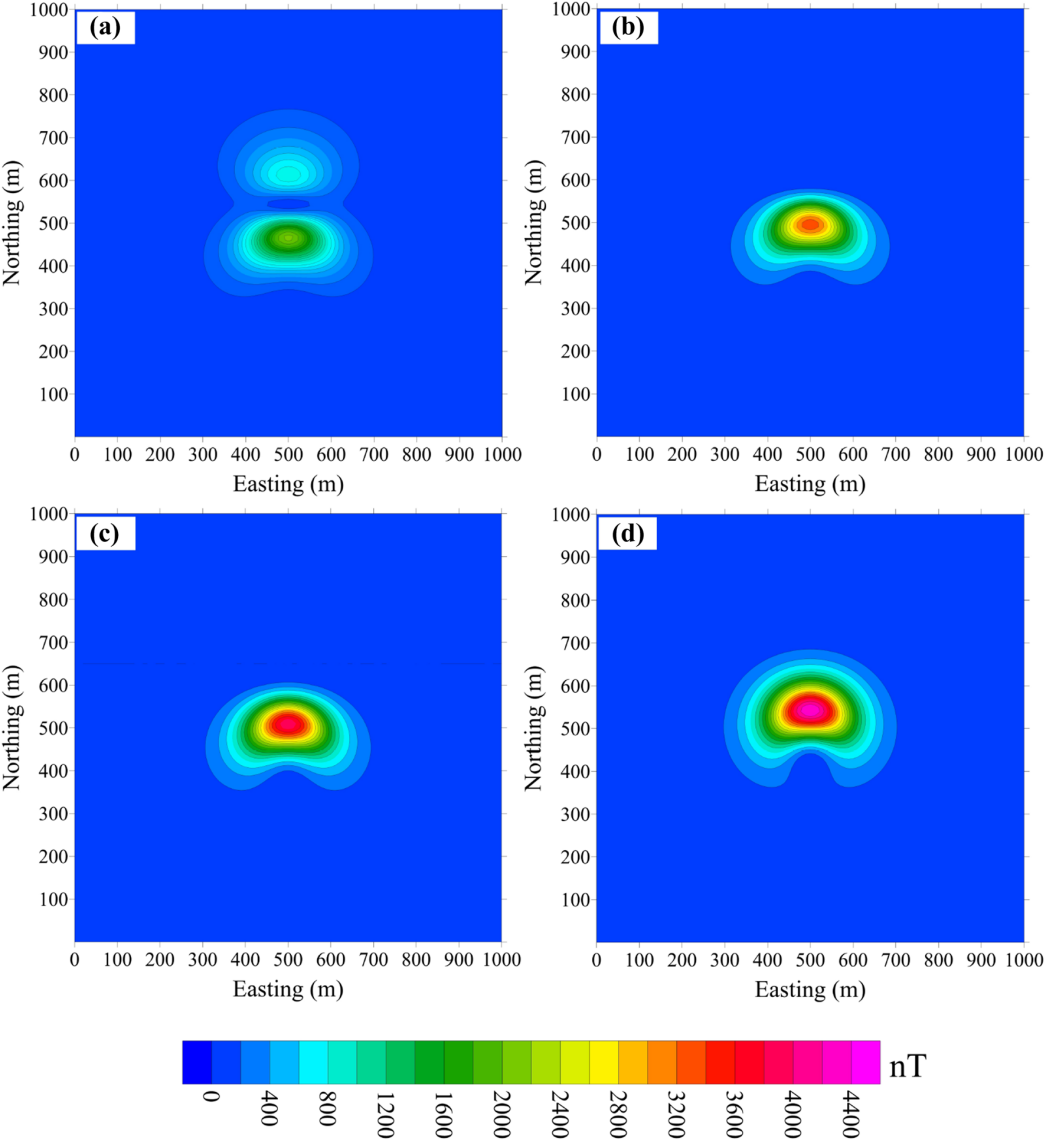
Difference between the projection anomaly and modulus difference anomaly with remanence. The total magnetization inclination is (**a**) 0°, (**b**) 30°, (**c**) 45° and (**d**) 90°.

Moreover, the error decreases when *I* changes from − 90° to 0° and then increases when *I* changes from 0° to 90°. The influence of *D* on the error is weak. It depends mainly on *I*, so we discuss the error caused only by the effect of *I*. In the cube model, *D* = 0° and *I* is set as 0°, 30°, 45° and 90°. We model the effect of *I* without and with remanence (Figs. 6, 7), respectively.

Figure 6a–d shows Δ*T*_*true*_ − Δ*T*_*pro*_ without remanence for *I* = 0°, 30°, 45° and 90°, respectively. The error distribution shows good symmetry with respect to the centre of the model, especially when the magnetization is horizontal or vertical. When the remanence is taken into account (*I*_*0*_ = 45°, *D*_*0*_ = 0°), the error pattern changes (Fig. 7) in both amplitude and shape.

As discussed above, the magnetic anomalies and directions affect the errors independently. However, the effect on Δ*T*_*true*_ − Δ*T*_*pro*_, which combined the amplitude and the direction of the magnetic anomaly, is unclear. Therefore, we use the trigonometric relationship between *T*, *T*_*0*_, *T*_*a*_ and *θ* to clarify this point. Equation (3) can be transformed into:

4 $$E = \Delta T_{true} - \Delta T_{pro} = \frac{{T_{a}^{2} - \Delta T_{true}^{2} }}{{2T_{0} }}.$$

**Figure 8:**
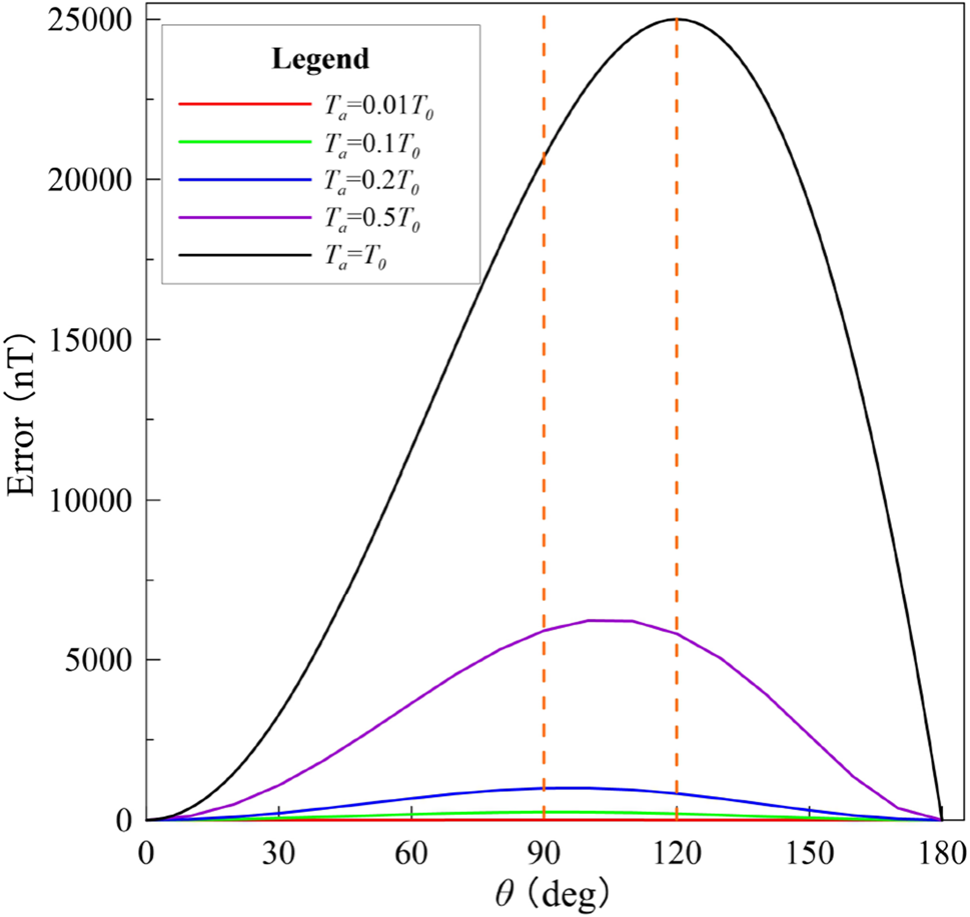
Difference between the projection anomaly and modulus difference anomaly for *θ* = 0°–180° is plotted for various values of the magnitude magnetic anomaly; *T*_0_ = 50,000 nT.

The *E* values for *θ* between 0° and 180° are plotted for various *T*_*a*_ values and *T*_*0*_ = 50,000 nT in Fig. 8. The *E* curves peak at 90° < *θ* < 120° and *E* increases as the magnetic anomaly increases. As Eq. (4) shows, the maximum error is reached when Δ*T*_*true*_ = 0 nT (see *E* in Table 1); in this case, the corresponding direction (θ*) meets the following relationship:

5 $$\begin{aligned} & \cos \theta^{*} = - {{T_{a} } \mathord{\left/ {\vphantom {{T_{a} } {(2T_{0} )}}} \right. \kern-\nulldelimiterspace} {(2T_{0} )}} \\ & \theta^{*} = \arccos \left( { - {\raise0.7ex\hbox{${T_{a} }$} \!\mathord{\left/ {\vphantom {{T_{a} } {2T_{0} }}}\right.\kern-\nulldelimiterspace} \!\lower0.7ex\hbox{${2T_{0} }$}}} \right). \\ \end{aligned}$$

**Figure 9:**
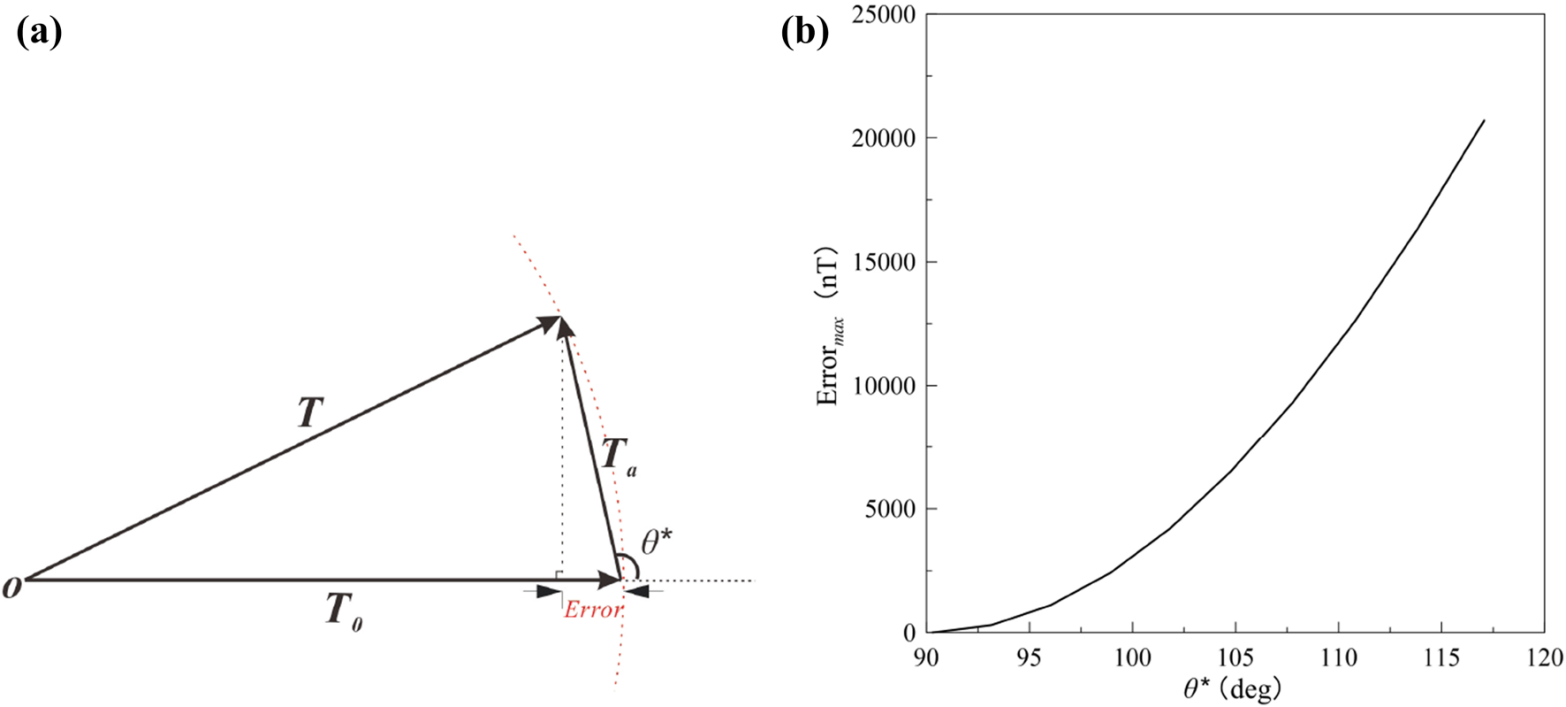
(**a**) Sketch of magnetic anomaly showing the geometry of maximum error when Δ*T*_true_ = 0 nT and (**b**) the relation between the maximum difference and its corresponding direction θ*.

The geometry of the three parameters is shown in Fig. 9a. The maximum error is plotted in Fig. 9b for θ* ranging from 90° to 120°.

Through the analysis above, we can find there is a huge error between Δ*T*_*true*_ and Δ*T*_*pro*_ in strong magnetic anomaly. Furthermore, it is more obvious that data-processing and interpretation, such as RTP, are affected by error. It takes disadvantage of our practical exploration. In addition, it is indicated that the error is enlarged and even doubled in the case of remanence. Indeed, most of the ore deposits exist the natural remanence. We should take the error seriously and use modulus difference anomaly rather than projection approximation for forward modelling to meet the needs of high-precision and quantitative process and interpretation in mineral exploration.

## Inversion of the projection and modulus difference anomalies

The direction of the normal geomagnetic filed is almost constant within a certain range, then it means the geomagnetic inclination *I*_0_ and declination *D*_0_ are certain. The magnitude magnetic anomaly *T*_*a*_ can be calculated from its three components (*H*_*ax*_, *H*_*ay*_ and *Z*_*a*_) in x-, y- and z-directions. At the same time, we assume that the components of the normal geomagnetic filed **T**_0_ in x-, y- and z-directions are *H*_*ox*_, *H*_*oy*_ and *Z*_*o*_. The components of **T**_0_ are fixed due to the geomagnetic direction is certain.

6 $$T_{a} = \sqrt {H_{ax}^{2} + H_{ay}^{2} + Z_{a}^{2} }$$

and

7 $$\begin{aligned} H_{ox} & = T_{0} \cos I_{0} \cos D_{0} \\ H_{oy} & = T_{0} \cos I_{0} \sin D_{0} \\ Z_{o} & = T_{0} \sin I_{0} \\ \end{aligned}$$

Then, the obtained anomaly **T** can also be translated to calculating the three components (*H*_*ox*_ + *H*_*ax*_), (*H*_*oy*_ + *H*_*ay*_) and (*Z*_*o*_ + *Z*_*a*_). Hence, Δ*T*_*pro*_ and Δ*T*_*true*_ can be expressed as:

8 $$\Delta T_{pro} = H_{ax} \cos I_{0} \cos D_{0} + H_{ay} \cos I_{0} \sin D_{0} + Z_{a} \sin I_{0}$$

and

9 $$\Delta T_{true} = \sqrt {(H_{ox} + H_{ax} )^{2} + (H_{oy} + H_{ay} )^{2} + (Z_{o} { + }Z_{a} )^{2} } - \sqrt {H_{ox}^{2} + H_{oy}^{2} + Z_{o}^{2} } .$$

The relationship between the components of magnitude magnetic anomaly *T*_*a*_ and magnetization intensity is linear.

10 $$H_{i} = \sum\limits_{j = 1}^{N} {G_{H(i,j)} m_{j} } = {\mathbf{G}}_{{{\mathbf{Hi}}}} {\mathbf{m}}.$$

*H*_*i*_ represents one of the three components (*H*_*ax*_, *H*_*ay*_ and *Z*_*a*_) of *T*_*a*_ at the *i*th observation point. **G**_**Hi**_ is the sensitivity matrix of *H*_*i*_, whose element is G_*H*(i,j)_. **m** is a matrix including the magnetization intensity of related model. *N* is the number of models. We assume that each mesh cell has a homogeneous magnetization intensity. Δ*T*_*pro*_ can be treated as a harmonic function, while Δ*T*_*true*_ is not an additive and harmonic function. Projection anomaly and modulus difference anomaly caused by the grid element of the *j*th unit magnetic intensity at the *i*th observation point can be shown as:

11 $$G_{pro(i,j)} = \frac{{\partial (\Delta T_{proi} )}}{{\partial m_{j} }} = G_{{H_{ax(i,j)} }} \cos I_{0} \cos D_{0} + G_{{H_{ay(i,j)} }} \cos I_{0} \sin D_{0} + G_{{Z_{a(i,j)} }} \sin I_{0}$$

and

12 $$\begin{aligned} G_{true(i,j)} & = \frac{{\partial (\Delta T_{truei} )}}{{\partial m_{j} }} \\ & = \frac{{(H_{ox} + H_{axi} ) \times G_{{H_{ax(i,j)} }} + (H_{oy} + H_{ayi} ) \times G_{{H_{ay(i,j)} }} + (Z_{o} + Z_{ai} ) \times G_{{Z_{a(i,j)} }} }}{{\sqrt {(H_{ox} + H_{axi} )^{2} + (H_{oy} + H_{ayi} )^{2} + (Z_{o} + Z_{ai} )^{2} } }}. \\ \end{aligned}$$

Apparently, the operator G_*true*(*i,j*)_ is complex because of the nonlinear relationship between the modulus difference anomaly with magnetization intensity; Δ*T*_*proi*_, Δ*T*_*truei*_, *H*_*axi*_, *H*_*ayi*_ and *Z*_*ai*_ are the predicted values at the *i*th observation point; **G**_***Hax***_, **G**_***Hay***_ and **G**_***Za***_ are the constant sensitivity matrices of *H*_*ax*_, *H*_*ay*_ and *Z*_*a*_, respectively. G_*Hax*(i,j)_ , G_*Hay*(i,j)_ and G_*Za*(i,j)_ are the elements of the matrices **G**_***Hax***_, **G**_***Hay***_ and **G**_***Za***_. **m** = (*m*_1_, *m*_2_, … *m*_j_ …, *m*_n_) is the model parameter vector to be solved and *m*_*j*_ represents the magnetic parameters of the *j*th mesh cell.

To solve the inverse problem, we minimize the following objective function:

13 $${\varvec{\upvarphi }}_{{\mathbf{d}}} = \left\| {{\mathbf{d}} - {\mathbf{Gm}}} \right\|_{2}^{2}$$

and

Subject to 0 < *m* < m_*Max*_.where **d** represents the geophysical observation data-vector, **G,** the *m* × *n*-dimensional sensitivity matrix, is a function operator and **m** represents the vector of magnetization intensity of the corresponding model.

14 $${\mathbf{G}}^{T} \Delta {\mathbf{d}} = {\mathbf{G}}^{T} {\mathbf{G}}\Delta {\mathbf{m}}.$$

The conjugate gradient method is one of the most effective methods for solving inversion problem and has been widely applied, such as geoelectrical^36–38^, gravity^39–42^ and magnetic^43,44^ data. The conjugate gradient method searches for the optimal solution after finite iteration convergence along the direction of the conjugate gradient. All the operations of the conjugate gradient method are vector operations. Variables can be used repeatedly during the cyclic iteration process, which reduces the operation time and storage space.

Inversion of potential data indeed faces serious non-uniqueness problem, which is a big challenge. There is no doubt that the non-uniqueness affects the accuracy and reliability of inversion results^45,46^. With *m* observation points data and the subsurface divided into n mesh cells, the proposed method has the similar degree of non-uniqueness as the traditional inversion methods^47–51^. The modulus difference anomaly inversion has the same unknow number of parameters (*n*) with the magnetization inversion. In other words, the non-uniqueness degree of the proposed method does not increase drastically compared with previous work. We add a simple magnetization intensity boundary constraint. Furthermore, we use the preconditioner instead of model constraint. The preconditioner^34^ plays the same role with depth weighting^52^, acting as a depth-weighted function in recovering the magnetization intensity distribution. It can also lower the condition number of coefficient matrix and increase the convergence rate.

15 $${\mathbf{P}}({\mathbf{G}}^{T} \Delta {\mathbf{d}}) = {\mathbf{P}}({\mathbf{G}}^{T} {\mathbf{G}}\Delta {\mathbf{m}}).$$

where the preconditioner ${\mathbf{P}} = z^{\beta } {\mathbf{I}}$. *z* is the depth of divided cell and *β* is a constant. The proper value *β* ranges from 4 to 6 in 3D inversion pointed by Liu^34^. It is concern with the distance between observation and mesh cell. **I** is a unit matrix. The preconditioned conjugate gradient algorithm follows that of Liu and Hu^53^ . First, the initial model (*m*_*0*_) is given. An appropriate model is of advantage to reduce the uncertainty. Then, the correction (Δ*m*) of the model’s parameters is calculated. The model’s parameters are updated by solving Eq. (13) with the preconditioned conjugate gradient method. The optimal solution is obtained after repeated iterations.

## Synthetic examples

### Cuboid model

**Figure 10:**
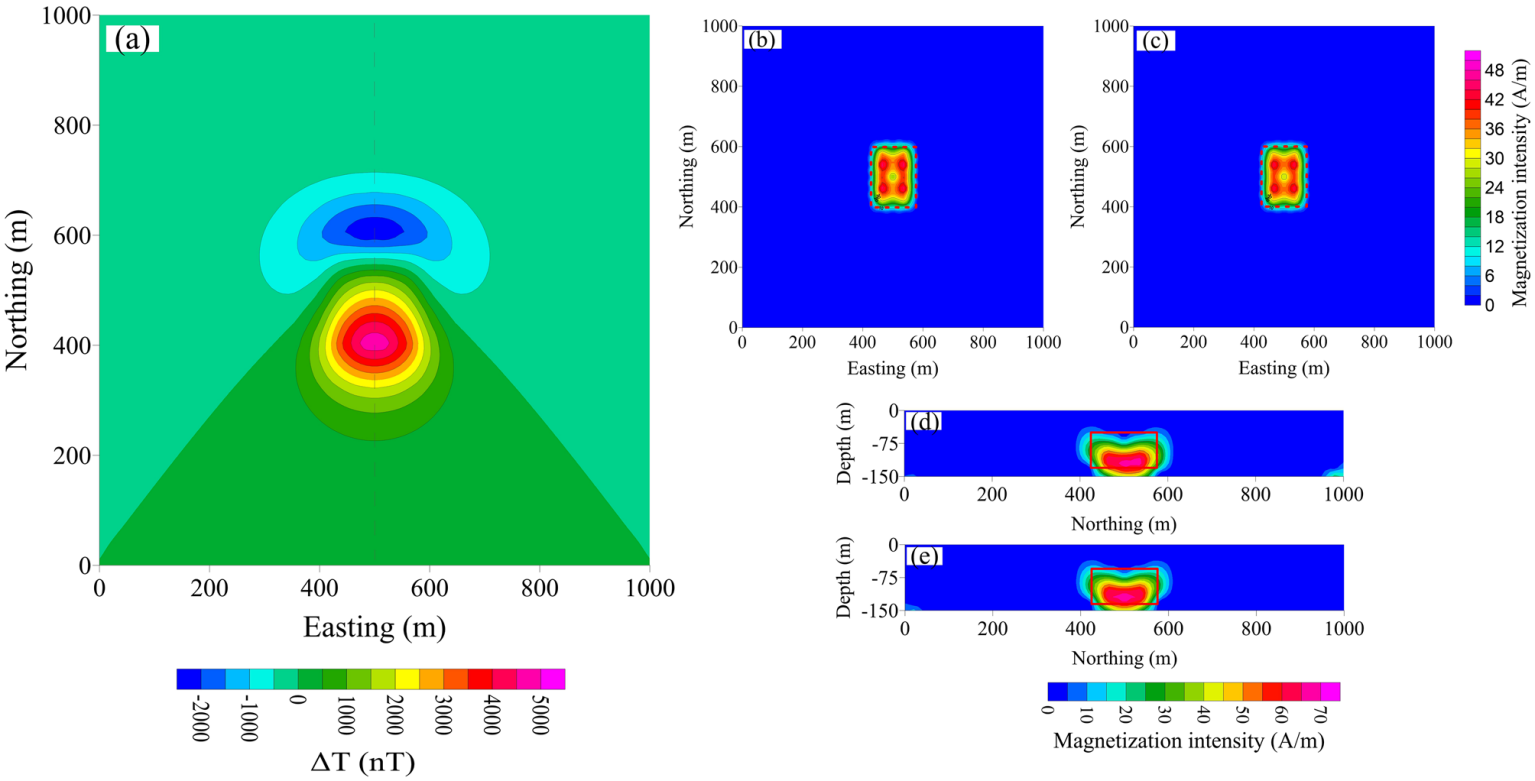
(**a**) Moderate-amplitude magnetic anomaly and its inversion from the projection anomaly data (**b**, **d**) and the modulus difference anomaly data (**c**, **e**); (**b**) and (**c**) show the horizontal cross-section at depth = 90 m; (**d**) and (**e**) show the vertical cross-section at easting = 500 m.

**Figure 11:**
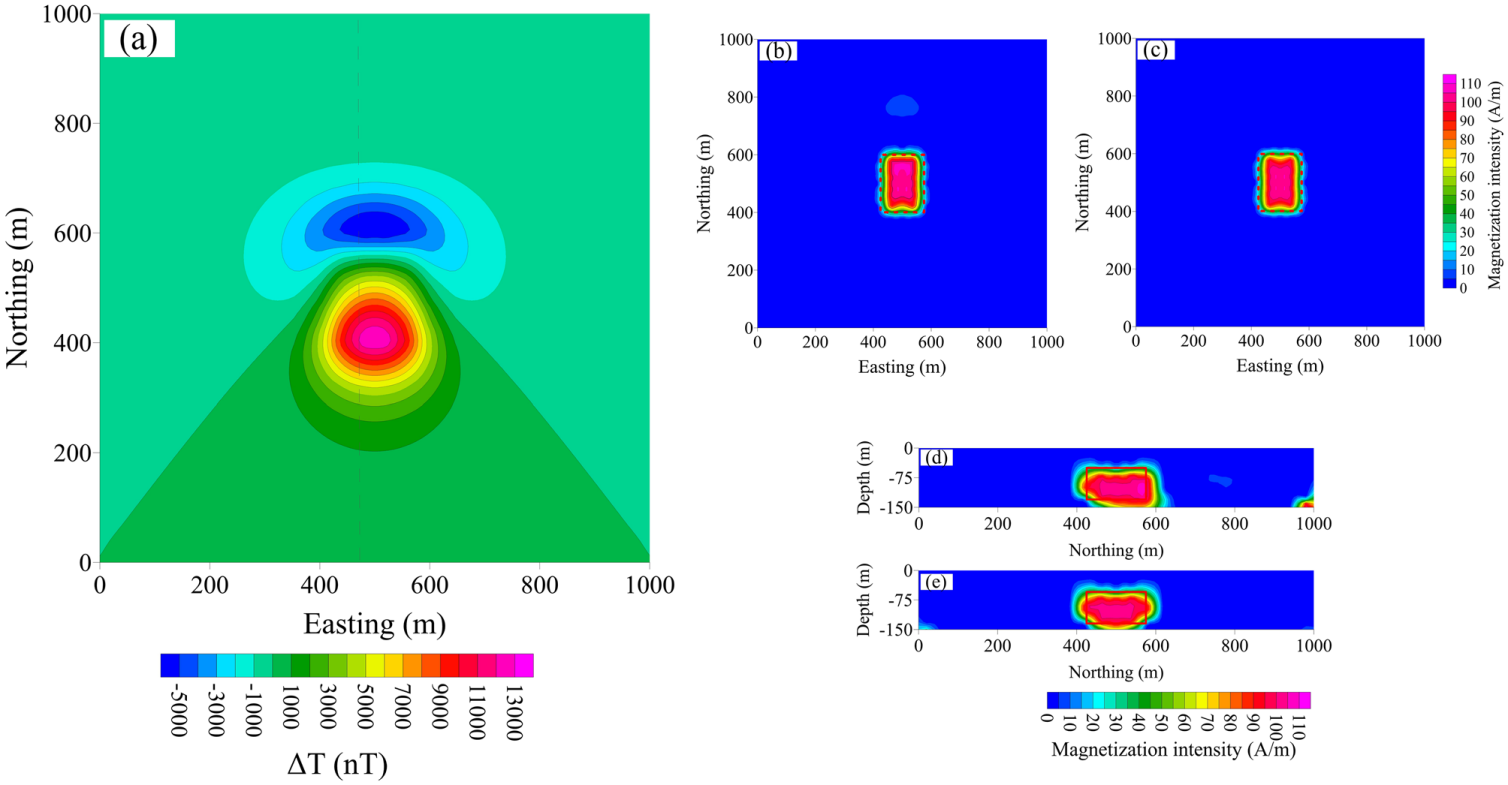
(**a**) High-amplitude magnetic anomaly and its inversion from the projection anomaly data (**b**, **d**) and the modulus difference anomaly data (**c**, **e**). (**b**) and (**c**) show horizontal cross-section at depth = 90 m; (**d**) and (**e**) show the  vertical cross-section at easting = 500 m.

In this experiment we conducted two tests—one with moderate and one with high magnetization intensity. The source was a cuboid model, 200 m × 150 m × 80 m, with its top buried at a depth of 50 m. The total inclination and declination were 45° and 0°, respectively. Figures 10a and 11a show the magnetic anomalies computed for *m* = 80 A/m and 110 A/m, respectively; the amplitudes of the total-field magnetic anomalies were 4,691 nT and 12,633 nT, respectively. Here, the magnetic anomaly is not displayed by the projection of the magnetic anomaly vector on the geomagnetic field but by the modulus difference.

The inversion results for *m* = 80 A/m are shown in Fig. 10b–d. Figure 10b, c shows the horizontal cross-section at a depth of 90 m calculated using Δ*T*_*pro*_ and Δ*T*_*true*_, respectively. Figure 10d, e shows the vertical cross-section at easting = 500 m calculated using Δ*T*_*pro*_ and Δ*T*_*true*_, respectively. There is no significant difference between the inversion results calculated by the two methods.

The inversion results for *m* = 110 A/m are shown in Fig. 11. The maximum misfit data of Δ*T*_*true*_ is 36 nT and the maximum misfit data of Δ*T*_*pro*_ is 159 nT. Figure 11d, e shows the vertical cross-section at easting = 500 m calculated using Δ*T*_*pro*_ and Δ*T*_*true*_, respectively. Figure 11b, c shows the horizontal cross-section at a depth of 90 m calculated using Δ*T*_*pro*_ and Δ*T*_*true*_, respectively. The red lines show the position of the true model. Here, the differences between the two datasets are clear. The inverted magnetization intensity distributions based on Δ*T*_*true*_ (Fig. 11c, e) are in good agreement with the true model and well describe the boundary of the target body. The inversion result shows it has favourable symmetry capability. However, the results based on Δ*T*_*pro*_ (Fig. 11b, d) are distorted. The results of the northing end show some fake anomalies at cross-section. The inverted magnetization intensity boundary is distorted; hence the recovered depths do not agree with the true depths. The inversion result based on Δ*T*_*true*_ are much closer to the source than those based on Δ*T*_*pro*_.

### The dipping dike model

**Figure 12:**
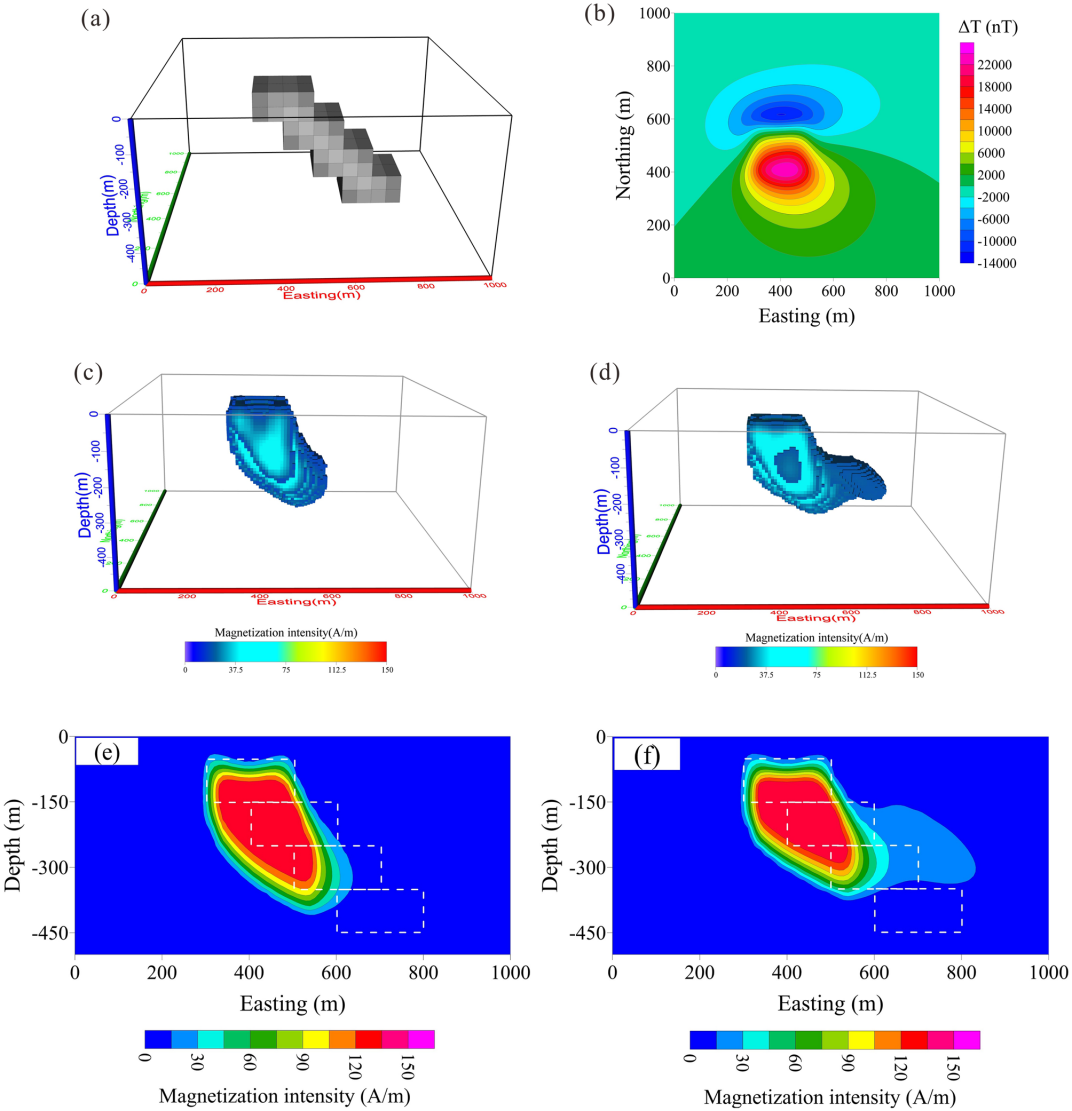
(**a**) The 3-D dipping dike model with (**b**) its observed magnetic anomaly and the 3D inversion results from (**c**) the projection anomaly and (**d**) modulus difference anomaly. The vertical cross-section map at northing = 500 m of the 3D inversion results from (**e**) the projection anomaly and (**f**) modulus difference anomaly data.

The dipping model is shown in Fig. 12a. Without considering the magnetic remanence, the total inclination and declination of the magnetization vector are 45° and 0°, respectively. The total magnetization intensity was set as 150 A/m and the geomagnetic field is 50,000 nT. The observed data are arranged in a 10 m × 20 m grid comprising 5,151 cells (Fig. 12b). The subsurface is divided into regular cells of size 25 × 25 × 25 m in the X-, Y-, Z-directions. The inversion results are shown in Fig. 12c–f. Figure 12c, d represents the 3-D inversion magnetization intensity distribution based on Δ*T*_*pro*_ and Δ*T*_*true*_, respectively. Figure 12e, f shows the vertical cross-section map at northing = 500 m derived from the 3-D inversion calculated using Δ*T*_*pro*_ and Δ*T*_*true*_, respectively.

Figure 12c, e shows the magnetization intensity distributions by calculated using Δ*T*_*pro*_. The inverted results show the target has a buried inclined geological structure. However, it differs from the true model, which we can see in the cross-section and 3D result. The map of the vertical cross-section at northing = 500 m (Fig. 12e) shows there is no magnetization at easting of 600–800 m with the depth of 350–450 m. it results in a significant discrepancy between the shapes of the inversion results and the model. Conversely, the boundary of the dipping dike is well displayed by magnetization intensity distributions of Δ*T*_*true*_ compared with those of Δ*T*_*pro*_. The inversion results based on Δ*T*_*true*_ accurately reproduced the shape of the source (Fig. 12d, f). and were generally consistent with Fig. 12a except for a little deviation at the depth 350–450 m caused by the deeply buried magnetic source. It is a common scene for inverted property to have a trend concentrating near the surface in potential field.

In terms of shape recovery and characterization of targets, modulus difference anomaly has advantages, which can better describe boundary of magnetic bodies at the same situation. It can show some characteristics of the model and realistically restore the shape of the model. These results show a marked improvement over the Δ*T*_*pro*_ inversion results in the intensity, shape and position of the source.

## Field example

**Figure 13:**
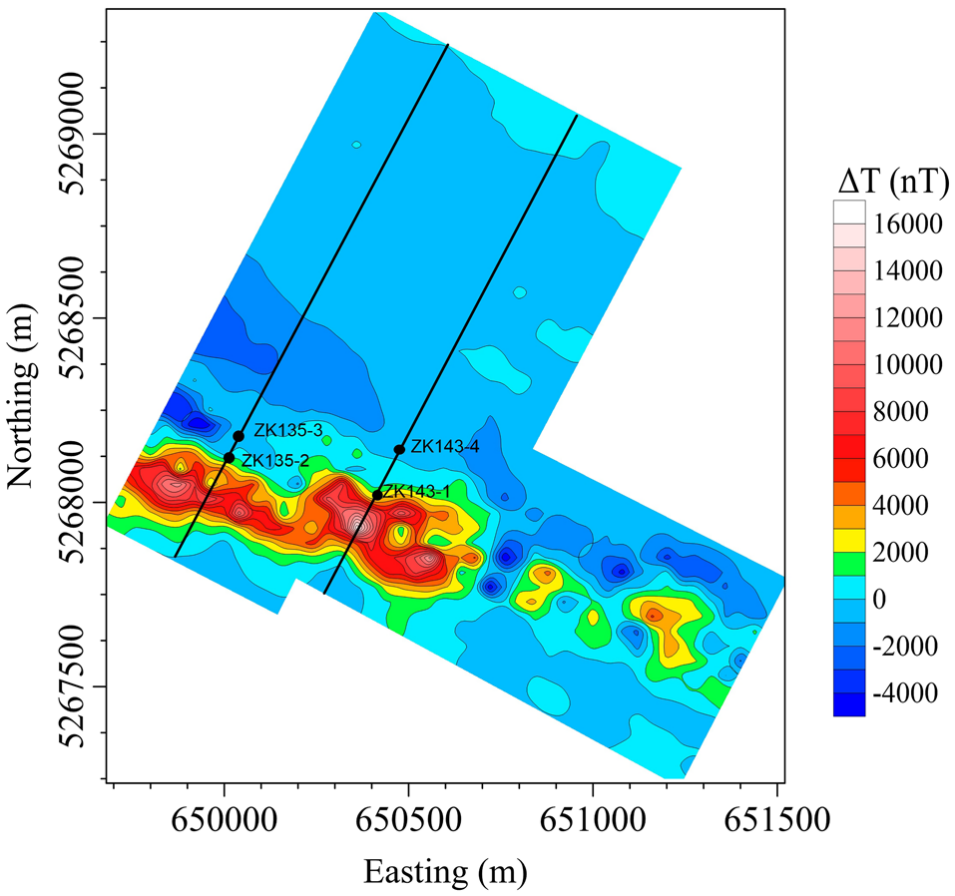
Observed modulus difference anomaly of Mengku iron-ore deposit in Xinjiang province, NW China.

**Figure 14:**
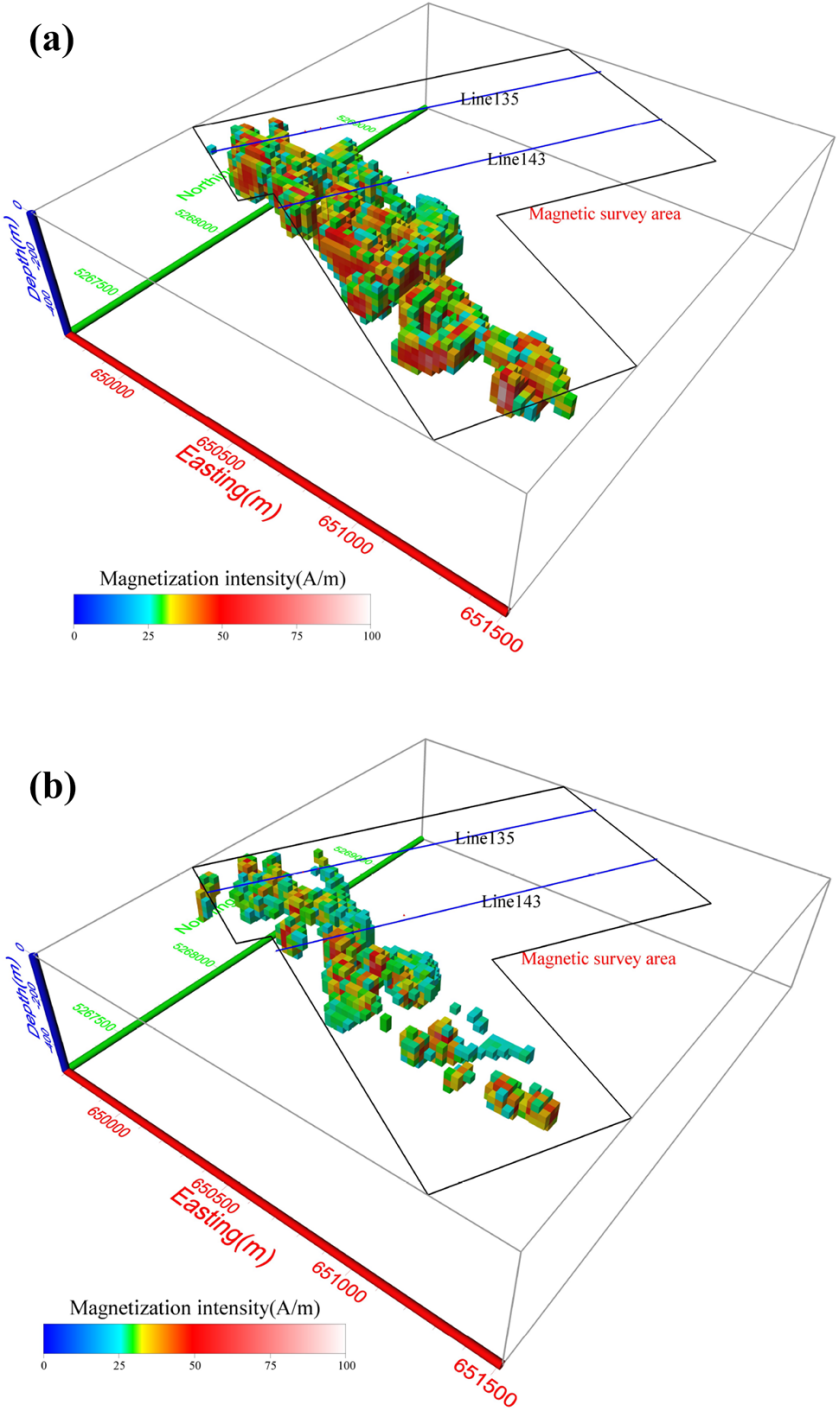
The 3D magnetic anomaly inversion results of the ore body shown in Fig. 13 calculated from (**a**) modulus difference anomaly data and (**b**) projection anomaly data.

The Mengku iron deposit is one of the largest iron deposits in Xinjiang Province, China. It is located in the Aletia active margin of the Siberian plate and the central region of the north-eastern margin of the Late Palaeozoic Maizi inland rift basin. The geomagnetic field intensity is *T*_*0*_ = 58,110 nT, with a geomagnetic inclination *I*_*0*_ = 67° and declination *D*_*0*_ = 3°. The main rock is amphibole oligo gneiss formed by regional metamorphism, which is locally known as fault migmatite. It is a polygenetic composite iron deposit formed by layered deposition, metamorphism and magmatic hydrothermal superimposition. Magnetite is the main mineral resource in the Mengku deposit. The rich magnetite has a strong magnetism with the susceptibility of 0.8–1.8 SI and the magnetization intensity is ranged from 40 to 90 A/m^35,54^. The magnetite ores show an amplitude change of − 6,000 nT from 22,749 nT, which provides data that are very suitable for testing inversion methods. The total-field magnetic anomaly is shown in Fig. 13. There are also some drillholes in line 135 and 143. Additionally, studies have shown that Mengku iron ore has strong remanence; the ratio between the induced magnetization and remanence is about 2.3 and the average residual ratio of its surrounding rocks is about 2.8^35^. Remanent magnetization changes the direction of the total field magnetization. Therefore, we used the correlation between the total magnitude anomaly and RTP to obtain the average magnetization direction^55^, where *I* = 68° and *D* = 85°. After obtaining the total field magnetization direction, it is used in the inversion of both projection anomaly and modulus difference anomaly to reduce the effect by remanence. We recovered the 3-D total magnetization intensity distribution. The subsurface was divided into 50 × 50 × 50 regular cells, with all the cells having the same magnetization intensity. A boundary constraint for the magnetization intensity of 0 A/m ≤ M ≤ 100 A/m was applied in the inversion. The inversion results based on Δ*T*_*true*_ and Δ*T*_*pro*_ are shown in Fig. 14a, b, respectively.

The magnetization intensity calculated using the Δ*T*_*true*_ inversion mainly ranges from 30 to 80 A/m (Fig. 14a), which is in good agreement with the magnetic properties of field samples (from poor magnetite orebodies to rich magnetite orebodies)^54^. However, most of the inverted magnetization intensities based on Δ*T*_*pro*_ lie within the range of 25–50 A/m (Fig. 14b). This again demonstrates the advantage of using Δ*T*_*true*_ to calculate the inverted magnetization intensity distribution. Figure 14 shows that the Δ*T*_*true*_-based anomalies have almost continuous highly-amplitude values while the Δ*T*_*pro*_-based inversion results have intermittent low-amplitude magnetization. At the southeast end of the model, there is an anomaly of almost 6,000 nT (Fig. 13), which is not well reproduced by the Δ*T*_*pro*_ inversion (Fig. 14b).

**Figure 15:**
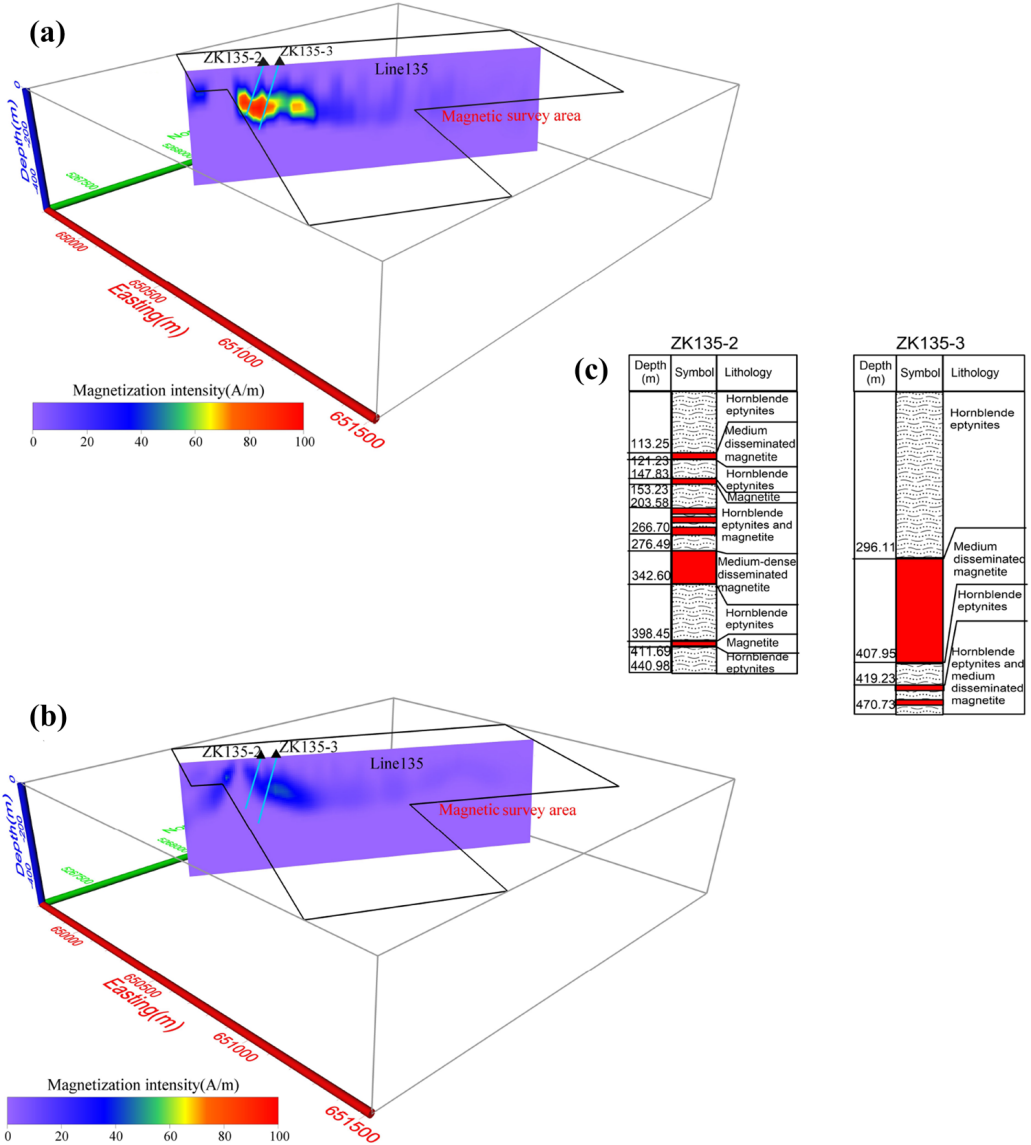
Vertical cross-section for Line135 of Mengku iron deposit: (**a**) directly inverting for modulus difference anomaly, (**b**) inversion from projection anomaly and (**c**) bore logs of drill holes.

**Figure 16:**
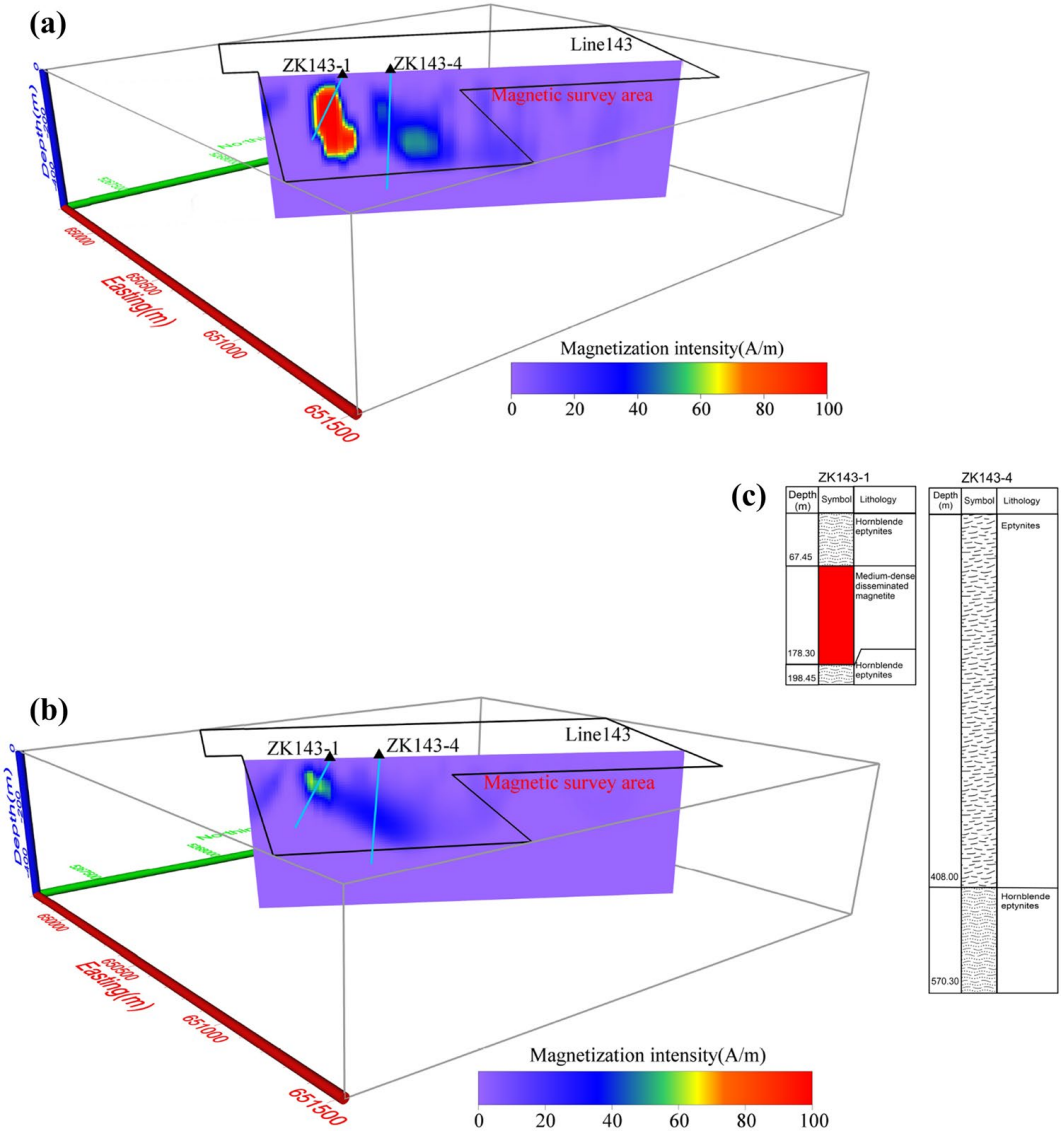
Vertical cross-section for Line143 of Mengku iron deposit: (**a**) directly inverting for modulus difference anomaly, (**b**) inversion from projection anomaly and (**c**) bore logs of drill holes.

According to the information from bore logs of drill holes, what’s more, we compare the difference inverting for between Δ*T*_*true*_ and Δ*T*_*pro*_ on the vertical cross-section between line 135 (Fig. 15) and 143 (Fig. 16). The results show inverting for Δ*T*_*true*_ (Fig. 15a), the presence of more than one magnetite belts in NE direction. However, the results from using Δ*T*_*pro*_ show significantly poor magnetization intensity (Fig. 15b), which is out of accord with the records of drillholes ZK135-2 and ZK135-3 (Fig. 15c). Thus, using Δ*T*_*pro*_ does not produce a good representation of the deposit shape and reserves, which leads to an inaccurate interpretation of the data. The drillhole ZK143-1 probes the magnetite belt at depth of 67–178 m and ZK143-4 do not intercept magnetite ores (Fig. 16c). The profile of line 143 inverted by Δ*T*_*true*_ (Fig. 16a) shows there are magnetite ores from 50 to 250 m with high susceptibility. Figure 16b shows the result using Δ*T*_*pro*_, the depth of the magnetite ores does not exceed 40 m, which is less than the results intercepted by drillholes. As discuss above, it indicated that directly inverting for Δ*T*_*true*_ produce more close results with the depth, shape and occurrences of mineral bodies. We therefore conclude that using Δ*T*_*true*_ in the inversion scheme to recover the magnetization distribution and location of orebodies will produce more reliable and acceptable results.

## Conclusions

For a strong magnetic anomaly, using projection anomaly to replace the modulus difference anomaly will yield errors. The error from using the projection anomaly as an approximation comes from two elements: the value of the magnetic anomaly and the magnetization direction. Our numerical modelling shows that the error increases exponentially as the magnetic anomaly increases, which is far beyond the precise magnetic allowance and no longer acceptable for high-precise interpretation; the influence of the magnetization direction on the error is mainly in the magnetic inclination, especially without remanence magnetisation. Furthermore, we found that when the error is the largest, the direction between the geomagnetic field and magnetic anomaly vector satisfies the mathematical relationship $\cos \theta^{*} = - T_{a} /2T_{0}$.

Through synthetic and field examples, we found that magnetic data inversion based on the projection anomaly do not produce a good fit with geological bodies in strong magnetic environments, while directly inverting for modulus difference anomaly data yield a more accurate representation of both the shape and location of the magnetic source, which is helpful to promote the horizonal and vertical resolution and reduce the ambiguity in the highly magnetic environment inversion.

As the requirements of higher accuracy in data processing and interpretation of magnetic sources are rising, the error of the magnetic anomaly should be reduced. Hence, using projection anomaly data is not appropriate. In cases of high-amplitude magnetic anomaly, we recommend using modulus difference anomaly data for reliable results.
